# Understanding COVID-19 Vaccine Effectiveness Against Death Using a Novel Measure: COVID Excess Mortality Percentage

**DOI:** 10.21203/rs.3.rs-2359020/v1

**Published:** 2022-12-16

**Authors:** Andy Yuan, Vladimir Atanasov, Paula Natalia Barreto Parra, Jeff Whittle, John Meurer, Benjamin Weston, Qian (Eric) Luo, Lorenzo Franchi, Ruohao Zhang, Bernard Black

**Affiliations:** Northwestern University; William & Mary; University of Illinois, Urbana-Champaign; Medical College of Wisconsin; Medical College of Wisconsin; Medical College of Wisconsin; George Washington University; Northwestern University; Centre College; Northwestern University

**Keywords:** COVID-19, COVID-19 mortality, cause of death, COVID Excess Mortality Percentage, vaccine effectiveness, vaccine efficacy, selection bias

## Abstract

COVID-19 vaccines have saved millions of lives and prevented countless adverse patient disease outcomes. Understanding the long-term effectiveness of these vaccines is imperative to developing recommendations for precautions and booster doses. Comparisons between more and less vaccinated groups may be misleading due to selection bias, as these groups may differ in underlying health status and thus risk of adverse COVID-19 outcomes. We study all adult deaths over April 1, 2021-June 30, 2022 in Milwaukee County, Wisconsin, linked to vaccination records, use mortality from other natural causes to proxy for underlying health, and report relative COVID-19 mortality risk (RMR) for vaccinees versus the unvaccinated, using a novel outcome measure that controls for selection effects. This measure, COVID Excess Mortality Percentage (CEMP) uses the non-COVID natural mortality rate (Non-Covid-NMR) as a measure of population risk of COVID mortality without vaccination. We validate this measure during the pre-vaccine period (r = 0.97) and demonstrate that selection effects are large, with Non-Covid-NMRs for two-dose vaccinees less than half those for the unvaccinated, and Non-COVID NMRs still lower for three dose (booster) recipients. Progressive waning of two-dose effectiveness is observed, with relative mortality risk (RMR) for two-dose vaccinees aged 60+ versus the unvaccinated of 11% during April-June 2021, rising steadily to 36% during the Omicron period (January-June, 2022). Notably, a booster dose reduced RMR to 10–11% for ages 60+. Boosters thus provide important additional protection against mortality.

## Introduction

COVID-19 vaccines have saved millions of lives, and mortality rates have fallen in 2022. However, U.S. mortality alone is still running at an annual rate of over 100,000. It is important to understand the mortality risk faced by the vaccinated, how protection varies with age, time since vaccination and other factors, and the benefit of booster doses.

Many studies have reported evidence on vaccine effectiveness (VE) against infection, hospitalization, and death (for succinctness, we cite primarily reviews).^1,2^ However, most of these studies face potential confounding due to selection bias. Most studies of effectiveness against mortality have limited controls for individual characteristics, often only age and gender,^3–9^ or study short time periods.^5,10–12^ U.S.-based studies typically lack population-level data,^7,13–15^ and datasets with information about vaccination status often have limited information about health status. Some use a test-negative design,^10,14,15^ which is prone to selection bias.^16^ If healthier people are more likely to be vaccinated, lower vaccinee mortality rates for vaccinees may in part reflect their (unobserved) better health and thus lower inherent risk.

In this study, we propose a novel method for evaluating COVID-19 vaccine effectiveness against mortality, which uses non-COVID natural mortality rates as a surrogate for underlying health. This surrogate is attractive as non-COVID mortality, COVID mortality, and vaccination status are often available for the same population. We propose, as an outcome measure, the COVID Excess Mortality Percentage (CEMP), defined as COVID-19 deaths divided by non-COVID natural deaths, converted to a percentage. The CEMP denominator controls for differences in population health between two groups, such as vaccinated versus unvaccinated.

We provide evidence on selection bias, measure the magnitude of this bias, and validate the CEMP measure as a means of reducing selection bias. We then use CEMP as an outcome to measure VE against death for the entire adult population of a large Midwestern city. We compare relative mortality risk (RMR = 1-VE) for vaccinated versus unvaccinated, using this measure, to previous approaches that do not include robust adjustment for underlying health status.

## Data And Methods

We obtain linked, de-identified mortality and vaccination records for all adults aged 18 + in Milwaukee County, Wisconsin (adult population 722,000), for January 1, 2021, through June 30, 2022, including residence zip code, age at death, gender, race/ethnicity, education, income, marital status, veteran status, manner of death, and text fields for cause of death and conditions leading to death. We use text analysis to identify deaths due to COVID-19 versus other natural causes; this counts more COVID-19 deaths than relying on ICD-10 cause-of-death codes prepared by the National Center for Health Statistics (NCHS), see Appendix for details. We treat vaccination as effective against mortality beginning 30 days after receipt; this time period allows for a vaccine dose to become fully effective, as well as the typical several-week lag between infection and death. We exclude immune-compromised decedents.

The mRNA vaccines (Moderna, Pfizer) use two initial does; J&J uses one dose. We report results based on number of doses, thus treating one J&J dose as equivalent to one mRNA dose, but obtain similar results if we exclude J&J vaccinees.

We define CEMP, VE versus the unvaccinated, and relative mortality risk after vaccination (RMR) in each time period, within a population group, as:

$$CEMP=\frac{COVID\;deaths\;}{\;non\;-\;COVIDnaturaldeaths\;}$$

$$VE=\frac{\left(CEMP_{unvax\;}\;-\;CEMP_{vax}\right)}{CEMP_{unvax\;}}$$

$$RMR=1-VE=\frac{CEMP_{vax}}{CEMP_{unvax}}$$

RMR can be obtained by comparing mortality rates for both groups, or as an odds ratio from logistic regression for a population containing both groups. We also compute RMR and VE for two-versus-one-dose and three-versus-two-dose vaccinees. CEMP, VE and RMR involve ratios, so could be undefined if the denominator is zero; we did not have this issue with our data and population groups.

CEMP represents the odds, for natural-cause decedents, of dying from COVID-19 versus other natural causes. The ratio of CEMPs for two groups, such as vaccinated versus unvaccinated, is an odds ratio, obtainable from logistic regression. We both conduct simple comparisons of CEMP for two groups, defined by age, gender, and vaccination status, and conduct multivariate logistic regression analysis of the association between vaccination and RMR, in which we adjust for other variables, available from death certificates, that may be associated with mortality risk. The predictors in this analysis are age, age^2^, zip-code-level socio-economic status (zip-SES), gender, race/ethnicity, education level, marital status, and military veteran status.

We measure race/ethnicity as non-Hispanic White (“White”), Black, non-Black Hispanic (“Hispanic”) and Other (including Asian, Native American, and mixed race). We measure zip-SES using the Graham Social Deprivation Index, which we have found to perform well in other work.^17^ We estimate population for 2020 from the American Community Survey, measure the number of vaccinees; and assume other persons are unvaccinated. We measure the Non-COVID-19 natural mortality rate (Non-Covid-NMR or NCNMR) for a group as natural deaths divided by population.

CEMP treats the non-COVID natural mortality rate as a proxy for overall health of a given group, and thus the likelihood of mortality if not vaccinated. We assessed the validity of this approach out-of-sample, by studying the correlation in Indiana (a nearby state where we have mortality data), between natural mortality in April-December 2019 (pre-COVID) and COVID-19 mortality over April-December 2020 (same months during the pre-vaccine period) Using 2019 natural mortality (rather than 2020 non-COVID natural mortality) to predict 2020 COVID mortality avoids the mechanical correlation which could arise if COVID deaths were undercounted, or prior COVID infection leads to higher non-COVID mortality.

Our RMR estimates will be biased only if both: (i) COVID-19 mortality is undercounted, and (ii) the degree of undercounting differs systematically between vaccinated and unvaccinated persons. We assess condition (i) – whether significant undercounting exists – as follows. We use natural mortality in Wisconsin over 2017–2019 to predict non-COVID natural mortality in the same month in 2020, using linear extrapolation, and compare predicted to measured non-COVID natural mortality in 2020. We assess whether measured non-COVID natural mortality exceeds predicted mortality, either overall, or during periods of high COVID mortality. Even if undercounting exists, we have no reason to expect that condition (ii) holds, but cannot provide evidence on this with our data.

## Results

### Validating the CEMP Measure

**Figure 1:**
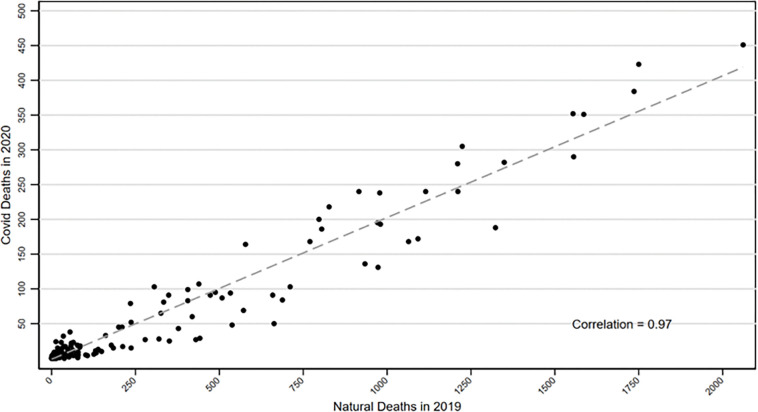
Out-of-Sample Correlation for Indiana between Natural Mortality in 2019 and COVID-19 Mortality in 2020. Figure shows scatterplot of natural mortality in Indiana over April-December 2019 against COVID-19 mortality over April-December 2020, for groups defined by age (18–39, 40–49, 50–59, 60–69, 70–79, 80–89, 90+)*gender*race/ethnicity*zip-SES quintile, best-fit regression line, and Pearson correlation coefficient. See Figure App-1 for similar in-sample scatter plots for Wisconsin and Milwaukee County

Figure 1 shows the correlation in Indiana between natural mortality in April-December 2019 (pre-COVID period) and COVID-19 mortality in April-December 2020 (COVID period, but pre-vaccine), for population groups defined by age (groups are 18–39, 40–49, 50–59, 60–69, 70–79, 80–89, and 90+, gender, race/ethnicity, and zip-SES. The Pearson correlation coefficient is 0.97, consistent with non-COVID natural mortality rates strongly predicting COVID mortality rates for unvaccinated persons.

Further validation comes from the multivariate regression analysis discussed below, in which RMR estimates within groups defined solely by age are similar to multivariate estimates that adjust for other factors that are associated with COVID-19 mortality. This suggests that the CEMP measure already controls well for population health.

### Validating the Text-Based Measure of COVID-19 Deaths

National evidence suggests undercounting of deaths attributable directly or indirectly to COVID-19.^18^ Bias in RMRs could result if COVID-19 deaths are undercounted and the degree of undercounting is associated with vaccination status. However, once we code COVID-19 deaths based on death certificate text fields, we find no evidence of important undercounting in Milwaukee or in Wisconsin generally.

For Milwaukee County, our text-based measure counts 1,911 COVID-19 deaths during our sample period; whereas ICD-10 codes from NCHS identify only 1,214 COVID-19 deaths (64% of those we include) (Appendix Table App-1). Most of the cases that we identify as COVID-19, but the NCHS does not, or vice-versa, are not close cases. For Wisconsin as a whole, the NCHS undercount is smaller; we count 12,595 COVID-19 deaths versus 11,512 with NCHS coding (92% of our count).

**Figure 2:**
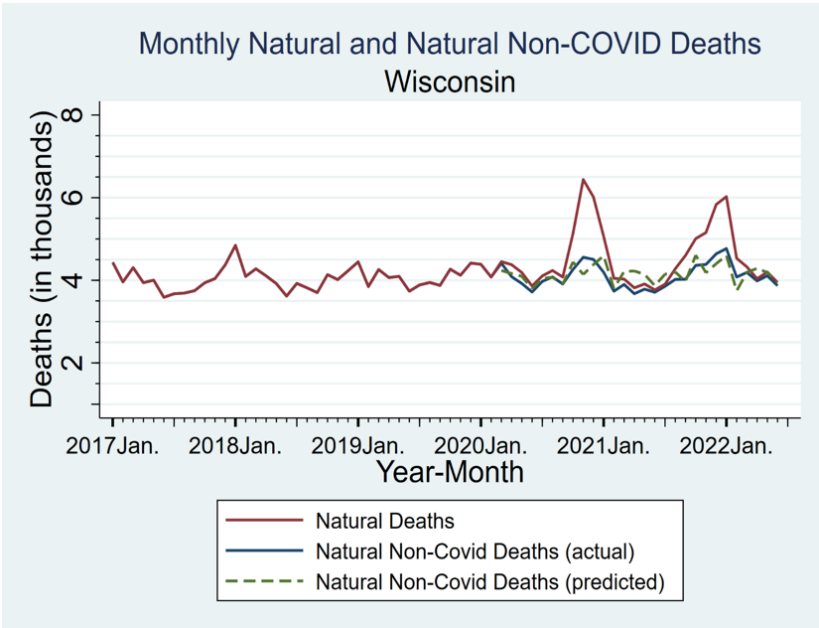
Actual versus Predicted Non-COVID Natural Mortality in Wisconsin Figure shows monthly data for natural non-COVID-19 and all natural deaths, for Wisconsin for January 2017 – June 2022. For the pandemic period starting March 2020, we show both actual and predicted natural non-COVID deaths. Predicted deaths are based on linear extrapolation from 2017–2019 to the same calendar month during the pandemic period. Natural deaths (including COVID-19 deaths) are shown as solid red line; this shows two large COVID-related peaks in late 2020 and late-2021-early 2022. Natural non-COVID-19 deaths (all natural deaths minus COVID-19 deaths) are shown as solid blue line. Predicted natural non-COVID deaths are shown as dashed green line.

Figure 2 reports monthly natural non-COVID-19 and all natural deaths for Wisconsin for 2017 – June 2022. For the pandemic period, we also show predicted natural non-COVID deaths, based on linear extrapolation from 2017–2019 to the same calendar month during the pandemic period. Natural deaths (including COVID-19 deaths) show two COVID-related peaks in late 2020 and late-2021 - early 2022. Natural non-COVID-19 deaths do not have corresponding spikes, which would be expected if COVID deaths were undercounted. Predicted natural non-COVID natural deaths (dashed line) are close to measured non-COVID natural deaths, sometimes higher or lower, but with no obvious pattern. Measured non-COVID natural deaths are never above the 95% confidence interval (CI) for the predicted deaths (for CIs, see Appendix Figure App-3).

This comparison, and the further analyses in Appendix Figures App-4 and App-5, provides evidence that our text-based coding of COVID-19 deaths does a good job of capturing actual COVID-19 deaths, and thus provides reasonable CEMP numerator and denominator.

### Evidence for Selection Effects

Table 1 provides evidence on differences in baseline health, using Non-Covid-NMR as a surrogate for health, between three-dose vaccinees, two-dose vaccinees, and the unvaccinated. The table reports Non-Covid-NMRs by age group, vaccination status, and time period, and the ratios of Non-COVID-NMR for 2-dose and 3-dose vaccinees to that for the unvaccinated. The table uses *’s to report statistical significance for the ratios, see Table App-13 for 95% confidence intervals (CIs). Vaccinees have substantially lower Non-Covid-NMRs, consistent with vaccinees being healthier on average, and thus likely facing lower baseline COVID mortality risk.

In the first two periods, when boosters were not available, NCNMR for ages 18–39 is 0.038% for the unvaccinated versus 0.008% for two-dose recipients (ratio of 4.75:1). For ages 40–59, unvaccinated NCNMR is 0.271% versus 0.099% for two-dose recipients (ratio of 2.74:1). For ages 60–79, unvaccinated NCNMR is 1.782% versus 0.577% for two-dose recipients (ratio of 3.09:1). Differences are smaller for persons aged 80+, but cumulative mortality is still 4.57% for unvaccinated versus 3.47% for 2-dose recipients (ratio of 1.31:1).

In the booster-available periods, there is a further separation, in which some persons who previously received two doses chose to obtain a third, booster dose, while others did not. This choice also involves strong selection effects. Three-dose recipients have lower Non-Covid-NMRs than two-dose recipients. This difference is largest for ages 60–79, for whom Non-COVID-NMR is 1.632% for two-dose recipients versus 0.797% for 3-dose recipients.

Appendix Figure App-2 provides information on Milwaukee County vaccination rates, which are broadly in line with national averages. Overall, around 74% of the adult population received at least one dose, 70% were fully vaccinated (one J&J dose or two mRNA doses), and of two-dose recipients, and 56% received a third dose with higher rates for older persons.

### CEMP and RMR by Time Period and Age Range: Overview

Table 2 reports the number of COVID-19 deaths, non-COVID-19 natural deaths, and CEMP (the ratio of the two), by age range and number of doses, for four periods: April-June 2021 (2Q-2021; Alpha as dominant variant); July-September 2021 (3Q-2021, Delta dominant, no boosters); October-December 2021 (4Q-2021, Delta dominant, boosters available); and January-June 2022 (1H-2022, Omicron dominant). These periods correspond to when the respective variants accounted for most infections (see Appendix)

Table 2 presents unadjusted results for COVID-19 deaths, other natural deaths, and CEMP by age group, time period, and vaccination status, and RMR for vaccinated versus unvaccinated. We present results by period, given evidence from other studies on waning vaccine effectiveness over time, differences in variant severity, and potential RMR differences between variants. We also report full-sample RMRs for three-versus-two, three-versus-one, and two-versus-one dose. The table uses *’s to report statistical significance for the ratios, see Appendix Table App-for CIs.

CEMP levels for all groups were low in 2Q-2021 – a relatively low period for COVID-19 infections and deaths – but rose substantially after that. For all unvaccinated, CEMP by period was 5.0%; 17.9%; 35.0%; and 18.6%. During the Omicron period, CEMP levels fell much more sharply for persons aged 18–59 than for ages 60+.

### RMR for Two Doses Versus the Unvaccinated

Two-dose RMR levels by period rose from 11%, to 17%, 21%, and 36%, implying decreased vaccine protection.

We find important differences in two-dose RMR for younger versus older persons. There was progressive waning for ages 60+, with RMR rising from 11–21%, 28%, and 34% by time period. For persons aged 18–59, RMR by period was 0% (no deaths), 6%, and 3%, before rising to 43% in the Omicron period. Two-dose RMR was nearly zero for ages 18–49, with only one death – a severely comorbid 35-year-old woman during 1Q-2022.

### RMR for Booster Dose

A booster dose offered considerable additional protection, especially for ages 60+. RMR for booster recipients aged 60 + was 10% in 4Q-2021 and 11% in 1H-2022. Booster protection varied with age; for ages 18–59, booster RMR was 0% (no booster-recipient deaths vs. 100 unvaccinated deaths). Older persons, in contrast, had meaningful RMRs after booster, although much lower than their two-dose RMRs.

**Figure 3:**
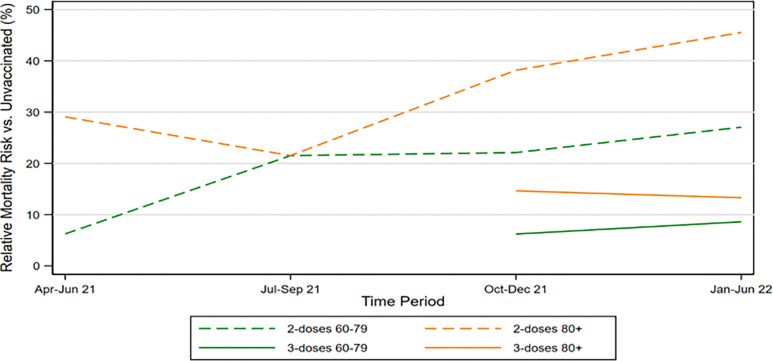
RMR for Two-Dose and Three-Dose Elderly Vaccinees Figure shows two-dose and three-dose relative mortality risk (RMR) versus the unvaccinated, by time period, for persons aged 60–79 and aged 80+.

Figure 3 shows RMR for two-dose elderly vaccinees by time period, and also RMR for three-dose vaccinees for the periods in which boosters were available. It includes separate lines for ages 60–79 and 80+. The figure shows both rising RMR over time (waning vaccine effectiveness) for both groups, and the higher RMR for ages 80+.

### RMR for One Dose

One-dose RMR versus the unvaccinated has been rarely studied. RMR relative to unvaccinated is substantial, at 57%, 58%, 29%, and 43% across our four time periods. One-dose RMR was similar in older and younger individuals. One-dose RMR, unlike two-dose RMR, did not exhibit waning. Thus, over time, as the benefit from a second dose, in reducing RMR has been shrinking.

### Multivariate Estimates

In Table 3, we use a multivariate logistic model to predict RMRs for one-dose, two-dose, and three-dose vaccinees versus the unvaccinated. The multivariate model includes factors which are known predict COVID-19 mortality, including gender, race/ethnicity, education, and zip-SES.^19,20^ Including these additional predictors has little effect on RMR estimates. The multivariate RMRs are consistent with the rates presented in Table 2. For example, in 1H-2022 (Omicron period), multivariate RMR for all two-dose recipients versus the unvaccinated is 34%, versus 36% from Table 2.

### Robustness Checks

Results for CEMP and RMR are consistent across a series of robustness checks: if we include the immune-compromised (Table App-7); exclude them and define immune-compromised more broadly (Table App-8), exclude J&J vaccine recipients (Table App-9), or use a shorter, 14-day minimum to treat vaccination as effective against death (Table App-10). Results are similar across genders and for Whites versus non-Whites (Tables App-11, App-12).

In Appendix Table App-14, we estimate RMRs using the COVID-19 mortality rate as the outcome. Consistent with the importance of the selection effects reported in Table 3, RMRs are lower. For example, for ages 60+, two-dose RMR across the sample periods is (8%, 10%, 17%, 25%), versus our finding above (11%, 17%, 21%, 36%).

## Discussion

A central need, when estimating how COVID vaccination affects mortality, is to estimate the counterfactual: What would COVID-19 mortality have been for the vaccinated, if they had not been vaccinated? We use Non-Covid-NMR as a proxy for background mortality risk, and find important differences in background mortality risk between vaccinated and unvaccinated and between two-dose and three-dose recipients. By this measure, two-dose recipients are healthier (have lower non-COVID-NMR) than the unvaccinated, and three-dose vaccinees are healthier than two-dose vaccinees. These selection effects, unless controlled for (through our CEMP measure or in another way) can produce large biases in VE estimates.

Non-Covid-NMR does an excellent job of predicting COVID-19 mortality during the pre-vaccine period, out-of-sample in Indiana (Fig. 1). It performs similarly well in sample, both for Wisconsin as a whole, and for Milwaukee County (Appendix Figure App-1). This suggests that using CEMP as the outcome when measuring RMR provides a good estimate of the protective effects of vaccination relative to the counterfactual. The similarity between unadjusted RMR estimates (Table 2) and multivariate estimates (Table 3) provides further evidence that the CEMP denominator does a good job of controlling for underlying health and mortality risk.

The data on Non-Covid-NMR ratios in Table 1 can be used to assess the extent of selection bias: Assume counterfactually that vaccination was useless against Covid mortality. What RMRs would one estimate, controlling only for age group? Given the high correlation between Non-Covid-NMR and COVID-19 mortality for the unvaccinated, the RMR ratios of vaccinated to unvaccinated in Table 1 provide approximate answers to this question. For example, for ages 40–79 during the Omicron period, estimated three-dose RMR would be 35%, versus true RMR (no vaccine effect) was 100%.

### Advantages of the CEMP Measure

CEMP as a measure of COVID-19 mortality, has attractive features relative to other measures. It relies only on death certificates, which are available for all decedents, but addresses selection effects, by using non-COVID NMR to proxy for population health, which is otherwise difficult to observe. An alternative approach, controlling for comorbidities captured in electronic health records, faces important limitations: comorbidity data may not be fully reported, and, in the U.S., population-level data on comorbidities is not available. Even studies that control for comorbidities often examine only people who seek medical care for COVID-19 infection^[8, 20]^ This will miss the association among underlying health, who becomes infected, and infection severity.

Using data only on decedents also avoids the challenges in estimating the population at risk. Population statistics may undercount some groups because of non-participation in the Census or the American Community Survey, or inaccurate data. While race/ethnicity can be inaccurately captured in death-certificate data, it is unlikely that inaccuracies will differ systematically between those who die of COVID-19 versus other natural causes.

### Overview of Results: Substantial RMRs, Large Value for Boosters

Our analysis provides a number of insights for vaccine effectiveness against mortality and selection effects in who gets vaccinated. First, our two-dose RMRs versus unvaccinated are substantially higher (VE is lower) than in other studies. The higher RMRs reflect our use of CEMP to address selection bias, as well as continued vaccine waning in the Omicron period.

The studies covered by the available systematic reviews report lower two-dose RMRs, from 6–17%, as compared to this study.^1,2^ We found only one study that reports similar two-dose RMRs for the pre-Omicron, pre-booster period. This study finds an 18% RMR for fully vaccinated U.S. veterans (two mRNA or one J&J) for ages < 65 and 28% for ages 65 + .^14^ Likely not coincidentally, this study uses the rich VA data to control for an extensive set of comorbidities; it also finds that vaccinees have lower all-cause mortality. We found only one other U.S. study that reports RMR from linked, population-wide mortality and vaccination data. A study of Puerto Rico through mid-October 2021 (thus pre-Omicron and pre-booster), reports two-dose RMR after 144 days (longest period considered) of 14% for Pfizer and 7% for Moderna, versus 3% and 1% soon after vaccination.^8^ This study does not control for selection effects. We did not find studies of VE against mortality covering the Omicron period.

Second, we find substantial waning of two-dose protection against mortality, with two-dose RMR versus unvaccinated for ages 60 + increasing from 11% in 2Q-2021 to 34% in 1H-2022. This contrasts with prior studies, which typically report limited waning against severe disease and death.^1,2^ Note that we cannot separate the effects of waning over time from differences in protection against different virus variants.

Third, we find that two-dose RMR increases with age, but boosters provide substantial additional protection. This makes boosters especially important for the elderly, particularly those age 80+. For ages 60 + three-dose RMR is 10% in 4Q-2021 and 11% in 1H-2022, versus 28% and 34% for two-doses. At the same time, three-dose RMRs are higher than reported in prior booster studies,^10^ again showing the importance of controlling for selection effects. Nonetheless, three-versus-two-dose differences in RMRs for ages 60 + are large, at 18% in 4Q-2021 and 23% in 1H-2022. The reduction in RMR is even higher for ages 80+, at 32% for 1H-2022. In effect, the higher two-dose RMRs that we find leave more room for boosters to reduce mortality, even though we also find higher three-dose RMRs than prior research. Our evidence supports public health messaging and policy that encourages boosters for the elderly.

Fourth, we find stronger relative two-dose protection for ages 18–59 in 2021, compared to older persons in the pre-Omicron period, but not in the Omicron period. Absolute COVID mortality risk after two doses is smaller for younger persons, but boosters are highly effective in reducing that risk: We find zero deaths among younger three-dose recipients. Our results for the Omicron period contrast to the perception among many younger persons that two doses provide sufficient protection.

Fifth, we find that a single dose provides only moderate protection, with RMR versus the unvaccinated around 50%. However, this protection appears to be long-lived. Limited waning has been reported before for the single-dose J&J vaccine.^7,21^ We find similar results for one-dose mRNA recipients.

### An Opportunity for Targeted Booster Messaging

Evidence of vaccine waning first appeared in mid-2021, initially from Israel. Based on this evidence, Israel launched a booster campaign in late July 2021, which reached the whole population by the end of August.^22^ Other countries soon followed, relying in part in Israeli evidence that boosters added important value. In the U.S., however, FDA scientists publicly questioned the need for boosters.^23^ An advisory committee to the Food and Drug Administration (FDA) in September 2021 approved only a limited rollout to the elderly and persons at risk due to occupational exposure;^24^ similarly, an advisory committee to the CDC endorsed boosters only for the elderly.^25^

Two months later, the FDA and CDC approved boosters for all adults; although a CDC recommendation came only at the end of November, 2021.^26^ Public health messaging remained muddled, with the value of boosters “lost in the sea of changing recommendations and guidance,”^27^ Even today, U.S. booster percentages lag many other countries,^28,29^ and public knowledge of booster recommendations is limited.^30^ Our study provides strong evidence on booster value for ages 60+, which account for the vast majority of COVID-19 deaths, and nearly all vaccinee deaths.

### Toward Enhanced Public Reporting of COVID-19 Mortality

Although many public sources report data on COVID-19 deaths. None reports a comparison to other natural deaths. Reporting both COVID-19 and non-COVID natural deaths, and ideally CEMP (the ratio of the two) would provide valuable information on the risk of death from COVID-19 versus other natural causes. Reporting CEMP would show that the unvaccinated face substantial COVID-19 mortality risk, even at younger ages. This might make more salient the large reductions in mortality risk from vaccination, and the value of a booster dose. Reporting both COVID-19 mortality and other natural mortality could also focus attention to selection effects, and their importance when estimating vaccine effectiveness.

### Limitations

This study has important limitations. We study only mortality. COVID-19 mortality is uncommon for younger persons, which limits statistical power. Our data is only for Milwaukee County, which is racially, ethnically, and economically diverse, but its COVID-19 experience may not be representative of other areas.

We do not observe, and thus cannot control for, prior COVID-19 infection. Especially in the Omicron era, many people, both vaccinated and unvaccinated, have already been infected. For them, VE can be understood as measuring the extra protection from hybrid immunity (from prior infection plus vaccination) versus natural immunity alone (from prior infection).

We do not observe individual health characteristics, except through the limited lens of death certificates. There could be vaccinated-vs-unvaccinated differences that affect COVID-19 mortality, not reflected in Non-Covid-NMR.

The CEMP measure has several inherent limitations. Though likely a reasonable proxy for overall health, it does not consider behavioral or other differences between more and less vaccinated groups. Behavioral differences are likely to exist. For example. the unvaccinated or less-vaccinated, may believe COVID is less severe than the maximally vaccinated, and therefore take fewer precautions. Conversely, vaccinated persons may accept greater risks of becoming infected, because they believe they are protected against serious illness. Neither ours nor other VE studies can control for behavioral differences.

The CEMP measure implicitly assumes that COVID-19 infection does not meaningfully affect non-COVID mortality. Yet COVID-19 infection is known to predict higher post-infection mortality from other causes, at least in the near term.^31^ This will cause downward bias in CEMP values. If this bias is similar for the vaccinated and unvaccinated, RMR estimates should still be unbiased. The downward bias in CEMP could be larger for the unvaccinated, who will on average face more severe COVID-19. If so, then RMR estimates based on CEMP will be somewhat *below those* we would estimate if we could attribute to COVID-19 these extra natural deaths. However, any bias is too small to be visible in Fig. 2.

COVID-19 deaths could be underreported, but we coded COVID-19 deaths based on reading death certificates; this produced significantly larger counts than ICD-10 codes from the NCHS. Any remaining undercount appears small (Fig. 2).

This study assesses vaccine effectiveness only against mortality, not against other important adverse outcomes including hospitalization and long-term symptoms. A full understanding of vaccine effectiveness must include a broader range of outcomes.

## Conclusion

We use a novel outcome measure, CEMP, to study how vaccination affects COVID-19 mortality risk. This measure uses mortality from other natural causes to control for selection effects in who gets vaccinated. We find substantially lower non-COVID natural mortality risk for vaccinated than for unvaccinated persons. Thus, though vaccination provides very substantial protection against mortality, the vaccinated would likely face lower COVID-19 risk even if not vaccinated. After controlling for these selection effects, we find increasing two-dose RMR over time, and large differences in RMR after two doses between younger (age 18–59) and older (age 60+) people. These findings imply that boosters are highly important in reducing mortality, especially for ages 60+. The RMRs after two-dose vaccination, and the meaningful although smaller three-dose RMRs for ages 60+, imply that non-vaccine mitigation strategies remain an important tool in reducing mortality in vaccinated populations, particularly among the elderly.

## Figures and Tables

**Table 1.** Non-Covid Natural Mortality Rate (Non-Covid-NMR) by Age Group and Time Period

	Time period	April-June 2021 (Alpha)		Jul-Sep 2021 (Delta no Booster)		Oct-Dec 2021 (Delta, With Booster)			Jan-June 2022 (Omicron)		
Age	Measure	Unvax	2 doses	Unvax	2 doses	Unvax	2 doses	3 doses	Unvax	2 doses	3 doses
18–39	Non-Covid NMR	0.016%	0.001%	0.022%	0.007%	0.021%	0.008%	0.000%	0.031%	0.015%	0.008%
	NCNMR ratio to unvax		**5.4%** **		**34.4%** **		**36.9%** **	**0.0%** ^**na**^		**49.0%** *	**27.1%** **
40–59	Non-Covid NMR	0.133%	0.053%	0.138%	0.046%	0.155%	0.077%	0.056%	0.313%	0.149%	0.110%
	NCNMR ratio to unvax		**39.4%** ***		**33.2%** ***		**49.9%** ***	**36.4%** ***		**47.5%** ***	**35.0%** ***
60–79	Non-Covid NMR	0.852%	0.279%	0.930%	0.298%	0.984%	0.438%	0.198%	1.695%	1.194%	0.599%
	NCNMR ratio to unvax		**32.7%** ***		**32.0%** ***		**44.5%** ***	**20.1%** ***		**70.4%** ***	**35.4%** ***
80+	Non-Covid NMR	2.582%	1.598%	1.988%	1.875%	2.550%	2.467%	1.032%	3.848%	4.743%	4.085%
	NCNMR ratio to unvax		**61.9%** ***		**94.3%**		**96.7%**	**40.5%** ***		**123.3%** *	**106.2%**

Sample is same as Table 1. Table shows Non-COVID Natural Mortality Rate (NCNMR) and relative NCNMR versus the unvaccinated and for people vaccinated with 2 doses or 3 doses. NCNMR is defined as the number of Non-COVID-19 natural deaths occurring among persons within the indicated age groups with the indicated vaccination status over the indicated period, divided by the estimated population of people in the same age group, vaccination status, and time period. For NCNMR ratios, *, **, *** indicates p < .05, .01, and .001, respectively; significant results (at p < .05 or better) in **boldface**. Statistical significance cannot be assessed for the one cell with 0% NCNMR.

**Table 2.** Vaccine Effectiveness (VE) by Age Group and Time Period

Age		April-Jun 2021 (Alpha)			Jul-Sep 2021 (Delta no Booster)			Oct-Dec 2021 (Delta, With Booster)				Jan-Jun 2022 (Omicron)			
Group	Measure	0 doses	1 dose	2 doses	0 doses	1 dose	2 doses	0 doses	1 dose	2 doses	3 doses	0 doses	1 dose	2 doses	3 doses

18–39	Covid deaths	2	0	0	7	0	0	16	0	0	0	4	0	1	0
	Other natural deaths	31	4	1	32	7	9	26	3	11	0	32	6	18	4
	CEMP	6.5%	0.0%	0.0%	21.9%	0.0%	0.0%	61.5%	0.0%	0.0%	NA	12.5%	0.0%	5.6%	0.0%
	RMR vs. Unvaccinated		0.0%	0.0%		0.0%	0.0%		0.0%	0.0%	NA		0.0%	44.4%	0.0%

40–59	Covid deaths	7	3	0	31	5	1	57	7	2	0	23	2	6	0
	Other natural deaths	147	41	27	113	32	53	102	43	95	3	177	57	109	84
	CEMP	4.8%	7.3%	0.0%	27.4%	15.6%	1.9%	55.9%	16.3%	2.1%	0.0%	13.0%	3.5%	5.5%	0.0%
	RMR vs. Unvaccinated		153.7%	0.0%		57.0%	6.9%		29.1%	3.8%	0.0%		27.0%	42.4%	0.0%

60–79	Covid deaths	26	3	1	49	7	12	95	6	30	1	90	8	25	10
	Other natural deaths	367	114	226	297	76	338	266	74	380	45	416	74	427	537
	CEMP	7.1%	2.6%	0.4%	16.5%	9.2%	3.6%	35.7%	8.1%	7.9%	2.2%	21.6%	10.8%	5.9%	1.9%
	RMR vs. Unvaccinated		37.1%	6.2%		55.8%	21.5%		22.7%	22.1%	6.2%		50.0%	27.1%	8.6%

80+	Covid deaths	6	1	2	26	6	13	49	6	37	2	56	7	27	18
	Other natural deaths	273	87	313	189	59	439	226	68	447	63	307	75	325	742
	CEMP	2.2%	1.1%	0.6%	13.8%	10.2%	3.0%	21.7%	8.8%	8.3%	3.2%	18.2%	9.3%	8.3%	2.4%
	RMR vs. Unvaccinated		52.3%	29.1%		73.9%	21.5%		40.7%	38.2%	14.6%		51.2%	45.5%	13.3%

**Total 18–59**	Covid deaths	9	3	0	38	5	1	73	7	2	0	27	2	7	0
	CEMP	5.1%	6.7%	0.0%	26.2%	12.8%	1.6%	57.0%	15.2%	1.9%	0.0%	12.9%	3.2%	5.5%	0.0%
	RMR vs. Unvaccinated		131.9%	0.0%^**na**^		48.9%	**6.2%** **		**26.7%** **	**3.3%** ***	0.0%^**na**^		24.6%	42.7%	0.0%^**na**^

**Total 60+**	Covid deaths	32	4	3	75	13	25	144	12	67	3	146	15	52	28
	CEMP	5.0%	2.0%	0.6%	15.4%	9.6%	3.2%	29.3%	8.5%	8.1%	2.8%	20.2%	10.1%	6.9%	2.2%
	RMR vs. Unvaccinated		**39.8%** ***	**11.1%** ***		62.4%	**20.8%** ***		**28.9%** ***	**27.7%** ***	**9.5%** ***		**49.9%** *	**34.2%** ***	**10.8%** ***

**All**	**Covid deaths**	41	7	3	113	18	26	217	19	69	3	173	17	59	28
	**CEMP**	5.0%	2.8%	0.5%	17.9%	10.3%	3.1%	35.0%	10.1%	7.4%	2.7%	18.6%	8.0%	6.7%	2.0%
	**RMR (versus unvax)**		56.8%	**10.6%** ***		**57.8%** *	**17.3%** ***		**28.9%** ***	**21.1%** ***	**7.7%** ***		**43.2%** **	**36.2%** ***	**11.0%** ***

Table shows COVID deaths, natural non-COVID deaths, COVID Excess Mortality Percentage (CEMP), and relative mortality risk (RMR) for persons vaccinated with 1, 2, or 3 doses, versus the unvaccinated and those vaccinated with fewer doses. RMR for a given comparison of two groups by vaccination status is defined as the ratio of CEMP for group 1 to CEMP for group 2. Sample is adult decedents in Milwaukee County, Wisconsin, excluding immune-compromised persons. Due to the nature of the sample, CEMP ratios and RMRs for age ranges and for all persons are effectively weighted by mortality rates. For RMR ratios to unvaccinated, *, **, *** indicates p < .05, .01, and .001, respectively; significant results (at p < .05 or better) in **boldface**. Statistical significance cannot be assessed for cells with 0% RMR.

**Table 3.** COVID-19 Relative Mortality Risk (RMR) Calculated Using a Multivariate Logit Model

			1 Dose		2 Doses		3 Doses	
Sample	Period	Remaining Risk	Estimate	95% CI	Estimate	95% CI	Estimate	95% CI
**18–59**	Apr-Jun 2021	Vs. unvax	178.3%	[47.4%, 670.9%]	0%^na^		No booster	
	Jul-Sep 2021	Vs. unvax	38.5%	[14.3%, 104.1%]	**3.3%** **	**[0.3%, 36.3%]**	No booster	
	Oct-Dec 2021	Vs. unvax	**20.0%** ***	**[7.9%, 50.7%]**	**2.4%** ***	**[0.6%, 9.9%]**	0%^na^	
		Vs. 2 doses					0%^na^	
	Jan-Jun 2022	Vs. unvax	23.3%	[4.6%, 118.2%]	62.8%	[25.3%, 156.2%]	0%^na^	
		Vs. 2 doses					0%^na^	
**60+**	Apr-Jun 2021	Vs. unvax	35.5%	[11.9%, 105.7%]	11.7%***	[3.4%, 40.3%]	No booster	
	Jul-Sep 2021	Vs. unvax	60.1%	[31.7%, 114.0%]	21.7%***	[13.2%, 35.6%]	No booster	
	Oct-Dec 2021	Vs. unvax	**26.8%** ***	**[14.1%, 50.9%]**	**27.9%** ***	**[20.1%, 38.7%]**	**9.6%** ***	**[3.0%, 30.7%]**
		Vs. 2 doses					33.9%	[10.6%, 108.2%]
	Jan-Jun 2022	Vs. unvax	**49.4%** *	**[27.8%, 87.9%]**	**32.9%** ***	**[23.5%, 46.0%]**	**11.1%** ***	**[7.2%, 17.1%]**
		Vs. 2 doses					**31.5%** ***	**[19.1%, 51.9%]**
**All (18+)**	Apr-Jun 2021	Vs. unvax	55.2%	[24.6%, 123.8%]	**11.9%** ***	**[3.5%, 40.4%]**	No booster	
	Jul-Sep 2021	Vs. unvax	58.0%*	[34.1%, 98.6%]	**19.0%** ***	**[12.0%, 30.2%]**	No booster	
	Oct-Dec 2021	Vs. unvax	**26.5%** ***	**[15.8%, 44.3%]**	**21.9%** ***	**[16.2%, 29.5%]**	**8.2%** ***	**[2.6%, 25.9%]**
		Vs. 2 doses					33.5%	[10.5%, 107.1%]
	Jan-Jun 2022	Vs. unvax	**43.2%** **	**[25.4%, 73.6%]**	**34.2%** ***	**[25.0%, 46.8%]**	**10.3%** ***	**[6.7%, 15.8%]**
		Vs. 2 doses					**29.2%** ***	**[18.1%, 47.2%]**

Table shows the odds ratios from logit regressions for persons who died of natural causes, for different numbers of vaccine doses by quarter over April 2021 – June 2022. These odds ratios directly measure RMR. Odds ratios are from logit model of Prob(Covid-19 Death) = f(doses received, baseline is unvaccinated or two-dose vaccinated depending on the RMR being estimated), with controls for age, age^2^, zip-SES (measured in centiles), gender, race/ethnicity, education level, marital status, and military veteran status. 95% confidence intervals (CIs) are in parentheses. Sample excludes immune-compromised persons. Coefficients on covariates are suppressed. RMR equals the odds ratio on the respective vaccination status indicators relative to the baseline vaccination status. *, **, *** indicates p < .05, .01, and .001, respectively; significant results (at p < .05 or better) in **boldface** Statistical significance cannot be assessed for cells with 0% RMR.

## Data Availability

The linked mortality and vaccination data on which this study relies was obtained under a data use agreement with the Wisconsin Department of Health Services and cannot be publicly shared.
